# Heavy Metals and Metalloids As a Cause for Protein Misfolding and Aggregation

**DOI:** 10.3390/biom4010252

**Published:** 2014-02-25

**Authors:** Markus J. Tamás, Sandeep K. Sharma, Sebastian Ibstedt, Therese Jacobson, Philipp Christen

**Affiliations:** 1 Department of Chemistry and Molecular Biology, University of Gothenburg, Gothenburg S-405 30, Sweden; E-Mails: sebastian.ibstedt@cmb.gu.se (S.I.); therese.jacobson@cmb.gu.se (T.J.); 2 Department of Biochemistry, University of Zurich, Zürich CH-8057, Switzerland; E-Mails: s.sharma@bioc.uzh.ch (S.K.S.); christen@bioc.uzh.ch (P.C.)

**Keywords:** protein folding, protein aggregation, chaperone, protein degradation, protein, metal toxicity, metalloid toxicity, yeast

## Abstract

While the toxicity of metals and metalloids, like arsenic, cadmium, mercury, lead and chromium, is undisputed, the underlying molecular mechanisms are not entirely clear. General consensus holds that proteins are the prime targets; heavy metals interfere with the physiological activity of specific, particularly susceptible proteins, either by forming a complex with functional side chain groups or by displacing essential metal ions in metalloproteins. Recent studies have revealed an additional mode of metal action targeted at proteins in a non-native state; certain heavy metals and metalloids have been found to inhibit the *in vitro* refolding of chemically denatured proteins, to interfere with protein folding *in vivo* and to cause aggregation of nascent proteins in living cells. Apparently, unfolded proteins with motile backbone and side chains are considerably more prone to engage in stable, pluridentate metal complexes than native proteins with their well-defined 3D structure. By interfering with the folding process, heavy metal ions and metalloids profoundly affect protein homeostasis and cell viability. This review describes how heavy metals impede protein folding and promote protein aggregation, how cells regulate quality control systems to protect themselves from metal toxicity and how metals might contribute to protein misfolding disorders.

## 1. Introduction

Heavy metals comprise a loosely defined group of elements that include transition metals and some metalloids. These elements affect cells and living organisms in various ways; some heavy metals have essential functions (e.g., iron, zinc, copper, manganese) and are toxic only in an overdose, whereas others are xenobiotic and highly toxic (e.g., arsenic, cadmium, lead, mercury). Thus, all cells and organisms maintain metal homeostasis within physiological or sub-toxic levels, respectively, and utilize metal detoxification mechanisms [1,2]. The toxicity of a given metal depends on its physicochemical properties and ligand preferences. “Soft” transition metals, like cadmium and mercury, prefer sulfur as their ligand. “Hard” transition metals, like chromium and manganese, and the metalloids, arsenic, antimony and selenium, favor oxygen in their higher oxidation states and sulfur in their lower oxidation states. Lead, iron, cobalt, nickel, copper and zinc may use oxygen, sulfur or nitrogen as ligands [2,3]. At the cellular level, heavy metals and metalloids have been reported: (1) to generate reactive oxygen species and to elicit oxidative stress; (2) to cause DNA damage and/or impair DNA repair mechanisms; (3) to interfere with membrane function and nutrient assimilation; and (4) to perturb protein function and activity [3,4,5]. At the molecular level, our understanding of how heavy metals exert these toxic effects is still rudimentary. There is a general consensus that proteins are key targets of heavy metals. Metals interfere with the biological activity of native, folded proteins through diverse modes of interaction; they may: (1) bind to free thiols or other functional groups in proteins; (2) displace essential metal ions in metalloproteins; or (3) catalyze oxidation of amino acid side chains [3,4,6]. Recent studies have now revealed an additional mode of metal action that targets non-folded proteins. Heavy metals and metalloids have been shown to inhibit refolding of chemically denatured proteins *in vitro*, to interfere with protein folding *in vivo* and to cause the aggregation of nascent proteins in living cells [7,8,9,10]. By interfering with the folding of nascent or non-native proteins, heavy metals profoundly affect protein homeostasis and cell viability.

Proteins participate in virtually every biological process. To function, most proteins fold into a strictly defined 3D structure, their native conformation. Misfolded proteins are cytotoxic, as they may aggregate and/or interact inappropriately with other cellular components. Numerous neurodegenerative and age-related disorders are associated with protein misfolding and aggregation [11,12,13]. There is accumulating evidence that metals might enhance the aggregation propensity of disease-associated proteins and promote the progression of certain neurodegenerative diseases through largely unknown mechanisms [14,15,16,17,18,19]. Evolutionarily conserved protein quality-control mechanisms protect cells against the harmful accumulation of protein aggregates. These quality-control systems consist of: (1) molecular chaperones that assist the folding of proteins into their functional conformation or rescue misfolded proteins by partial unfolding, thereby giving them another chance to assume their native structure; and of (2) protein degradation pathways, including the proteasome, as well as lysosomal and autophagic processes, which clear cells from misfolded and aggregated proteins. The malfunction of these quality-control systems may result in disease or cell death [11,20,21].

It is important to note that many proteins require metal ions for proper folding and/or for catalytic activity and that protein misfolding and aggregation might occur when the homeostasis of essential metal ions, such as iron, copper and zinc, is disturbed [19,22]. The mechanisms of (essential) metal homeostasis, metal sensing and metal coordination have been reviewed elsewhere [1,19,22,23,24,25]. Here, we review how heavy metals and metalloids, in particular arsenic, cadmium, mercury, lead and chromium, interfere with protein folding, how these agents promote protein aggregation and how quality-control systems protect cells from their toxicity.

## 2. Impact of Heavy Metals on Native, Correctly Folded Proteins

Heavy metals and metalloids can bind to native proteins and inhibit their biological activity. For example, methylmercury (MeHg) strongly inhibits the activity of yeast l-glutamine:d-fructose-6-phosphate amidotransferase, and overexpression of the enzyme confers MeHg resistance to yeast cells, suggesting that it is indeed targeted by MeHg [26]. How MeHg inhibits this enzyme has not yet been ascertained. Cadmium inhibits human thiol transferases (glutathione reductase, thioredoxin reductase, thioredoxin) *in vitro*, possibly by binding to cysteine residues in their active sites [27]. Inhibition of thiol transferases probably leads to increased oxidative stress and cell damage. Cadmium may also displace zinc and calcium ions from metalloproteins and zinc finger proteins [28,29]. It is not known, however, to what extent this metal exchange contributes to cadmium toxicity. Cadmium has been shown to cause DNA damage by targeting the DNA mismatch repair system [30]. Specifically, cadmium inhibits the ATPase activity of the Msh2p-Msh6p complex [31], but it is not known whether it binds to a specific site or displaces a critical zinc ion. Trivalent arsenic (arsenite, (As(III))) interacts with many proteins [32] and is supposed to interfere with their activity, e.g., binding to β-tubulin inhibits its polymerization [33]. Likewise, arsenic trioxide (ATO), the form of arsenite used in cancer therapy, inhibits mammalian thioredoxin reductase (TrxR), probably by directly binding to the enzyme. TrxR inhibition leads to the oxidation of thioredoxin, which is one of the main thiol-dependent electron donor systems in mammalian cells, thereby affecting the cellular redox environment and a wide range of cellular processes [34]. ATO directly binds to cysteine residues in the PML-RARα oncoprotein that is expressed in patients suffering from acute promyelocytic leukemia (APL). ATO-binding induces oligomerization and subsequent degradation of the aberrant PML-RARα fusion protein, which, in turn, inhibits the growth of APL cells, thereby curing the disease [35,36]. In contrast, binding of arsenic to pyruvate kinase M2 does not impair the activity of this enzyme [33]. Moreover, in a human cell line, the pyruvate dehydrogenase multi-enzyme complex proved to be more sensitive to inhibition by arsenic-induced reactive oxygen species than to direct inhibition by arsenicals [37]. Apparently, certain native proteins are more susceptible to metal-induced oxidation than to direct metal binding.

## 3. Proteins Are Vulnerable during Folding, Be It *In Vitro* or *In Vivo*

Folding *in vitro* starts with a chemically denatured protein with a spatially undefined random coil structure and is initiated by dilution or removal of the denaturing agent, such as urea or guanidinium chloride. *In vivo* folding, however, is a cotranslational process, which starts as soon as an NH_2_-terminal segment of sufficient length has been synthesized and has left the exit tunnel of the ribosome. The first synthesized domain thus acquires 3D structural elements (α-helices, β-sheets and, in part, a tertiary structure in a highly motile molten globule state) while the rest of the polypeptide chain is still being synthesized on the ribosome [38,39].

Experiments on *in vitro* refolding are usually performed with a purified protein at low concentration, i.e., under conditions that maximize the yield of refolding. In contrast, *in vivo* folding occurs in the crowded interior of a cell at a protein concentration of 300–400 mg/mL, which greatly enhances the chances of intermolecular interactions, including the aggregation of still incompletely folded or misfolded proteins. The folding of many nascent polypeptide chains requires the assistance of molecular chaperones as soon as they emerge from the exit tunnel of the ribosome. In particular, molecular chaperones (heat shock proteins) of the Hsp70 family and chaperonins (Hsp60) are engaged. Chaperones improve the yield of folding of the nascent chains by two different mechanisms: on the one hand, they prevent hydrophobic intra- and inter-molecular contacts that might result in misfolding and aggregation; on the other hand, they rescue misfolded and aggregated proteins by unfolding and giving them another chance to proceed on their correct folding pathway. Irreversibly misfolded proteins are eliminated by quality control mechanisms [20,21]. Although the intracellular pathway of protein folding is considerably more complex than that of *in vitro* folding, both roads lead to the unique 3D structure of the given protein.

Intramolecular interactions within the backbone of the polypeptide chain and between its side chains are responsible for the stable and strictly defined 3D structure of a given protein. The final, functionally active conformation corresponds to the conformational state of lowest free energy and is determined solely by the amino acid sequence of the protein [40]. Hydrogen bonds as directed forces determine the spatial course of the polypeptide chain, and the hydrophobic effects of apolar side chains provide the thermodynamic stability of the 3D structure [41]. The individual molecules of a given polypeptide under given conditions (particularly pH, temperature and the type and concentration of salt ions) apparently attain their final 3D structure essentially via the same transition states and intermediates. The rate of folding depends on the particular protein and varies in a wide range. Small proteins (<100 residues) with a single folding domain may reach their final conformation within milliseconds, whereas larger multi-domain proteins may require minutes to hours, proline isomerization and disulfide isomerization being possible rate-limiting steps [42].

Experimental *in vitro* folding and physiological intracellular *in vivo* folding share, despite their differences, important features; not only an identical end product, the structure of which is determined and stabilized by identical interactions, but also intermediary states of folding in which segments of the backbone and the side chains are still motile and the functional groups of the side chains are not yet shielded from the solute. These features make both *in vitro* and *in vivo* folding proteins prone to interacting with heavy metal ions and metalloids.

## 4. Heavy Metals and Metalloids Interact with Functional Groups of Chemically Denatured Proteins and Inhibit Their *In Vitro* Refolding

Heavy metal ions, such as Cd^2+^, Hg^2+^ and Pb^2+^, are well known to form relatively labile monodentate and highly stable pluridentate complexes with sulfur, nitrogen and oxygen atoms of proteins. The values of the apparent dissociation equilibrium constants, *K_d_*’, of the respective monodentate complexes of these metal ions with thiol, imidazole and carboxyl groups, the three most important ligands for divalent metal ions in proteins, depend on the particular metal-ligand combination and vary over a very broad range from picomolar to millimolar (Table 1). Pluridentate (multidentate) complexes are much more stable, their *K_d_*’ values being close to the product of the *K_d_*’ values of all the participating monodentate interactions. Such highly stable pluridentate complexes, most of them with a tetrahedral (four ligands) or octahedral (eight ligands) geometry, are found in metalloproteins [43,44,45].

**Table 1:** Dissociation equilibrium constants of monodentate metal complexes.

	*K_d_’* at pH 7 *			Approximate pK_a_ in proteins
	Cd^2+^	Hg^2+^	Pb^2+^	
Thiol group	2.5 µM	0.063 nM	13 µM	9.4
Imidazole group	2.0 mM	200 µM	6.3 mM	6.5
Carboxyl group	16 mM	2.5 µM	13 mM	4.6

* *K_d_’*is the apparent dissociation equilibrium constant at pH 7 of the reaction ML
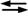
M + L, where ML is the 1:1 complex of the metal ion (M) and ligand (L) [45].

The recent observations that Cd^2+^, Hg^2+^, Pb^2+^ and arsenite effectively interfere with the refolding of chemically denatured proteins has opened up a novel perspective in metal toxicology [6,7,8,9]. Their inhibitory effect on the refolding of chemically denatured proteins becomes apparent without delay after addition at nanomolar final concentrations. In contrast, native proteins are much less affected by metal ions under the same conditions, being inactivated only to a limited degree in a slow time-dependent process [7]. Dose-response curves for the inhibition of refolding, i.e*.*, for the decrease in the folding yield, indicate IC_50_ values in the two- to three-digit nanomolar range (Table 2). It is important to note that these IC_50_ values had to be determined at a protein concentration of 350 nM, because the reproducibility of measurements at lower concentrations had proven unsatisfactory. The IC_50_ values given in Table 2 are thus strictly apparent values obtained at metal/metalloid concentrations lower than the protein concentration; they perforce underestimate the folding-inhibitory effect of the metal ions. The example of glucose-6-phosphate dehydrogenase, a cysteine-free enzyme, shows that stable chelate-like structures that impede refolding apparently are also formed in proteins lacking cysteine residues (Table 2).

Neither reduced glutathione nor the chelator EDTA rescue the protein that has misfolded in the presence of Cd^2+^. In contrast, the ATP-dependent Hsp70 chaperone system (DnaK/DnaJ/GrpE of *Escherichia coli* was used) significantly attenuates the folding-inhibitory effect of metal ions. Accordingly, ATP consumption measurements show an increased engagement of the chaperone system in the presence of metal ions; during *in vitro* refolding, metal ions almost double the chaperone load [7].

The ranking of the metal ions in terms of their efficacy in folding inhibition is Hg^2+^ > Cd^2+^ > Pb^2+^ (Table 2) and correlates with the relative stability of their monodentate complexes with thiol, imidazole and carboxylate groups in proteins (Table 1). The IC_50_ of both Cd^2+^ and Pb^2+^ are (despite being overestimated) two to three orders of magnitude lower than the dissociation equilibrium constants of their monodentate complexes with protein side chain groups (very tight binding of Hg^2+^ being an exception, probably due to its grossly underestimated inhibitory effect, i.e., much too high apparent IC_50_, as discussed above). The discrepancy indicates that highly stable pluridentate complexes rather than relatively labile monodentate complexes interfere with the proper refolding of the proteins. The chances of metal ions to interact with several potential ligands and to form stable pluridentate complexes with their metal-specific geometry [43,44,45] is of course much higher in the case of a denatured protein with motile random coil conformation and fully accessible side chain groups than in the case of a folded protein with its stable native structure and the side chains in a fixed and, for part of them, even buried location. In a folded protein, the formation of pluridentate complexes with specific geometry would require its partial unfolding. Compared with the rates of ribosomal protein synthesis (up to 20 residues per second in prokaryotes at 37 °C [46] and slower in eukaryotes [47]) and the rate of *in vitro* refolding, the formation of metal-protein complexes is extremely fast; the inner-sphere aquo ions of divalent metal ions are substituted with a rate in the range of 10^7^–10^9^ s^−1^ [2].

**Table 2:** IC_50_ values of Cd^2+^, Hg^2+^, Pb^2+^ and NaAsO_2_ for the inhibition of protein refolding.

	*IC*_50_ value (nM)			
	Cd^2+^	Hg^2+^	Pb^2+^	NaAsO_2_
**Luciferase**				
Spontaneous refolding	66 ± 11	40 ± 3	63 ± 6	426 ± 4
Chaperone-assisted refolding	100 ± 5	53 ± 2	140 ± 11	297 ± 13
Chaperone-mediated disaggregation	280 ± 4	210 ± 16	325 ± 11	613 ± 31
**Lactate dehydrogenase**				
Spontaneous refolding	68 ± 2	58 ± 6	74 ± 9	ND
**Malate dehydrogenase**				
Spontaneous refolding	300 ± 45	290 ± 16	520 ± 44	ND
**Glucose-6-phosphate dehydrogenase**				
Spontaneous refolding	340 ± 15	230 ± 18	>600	ND

* IC_50_ values with SEM from three independent experimental datasets [7,9]. The values given are apparent values determined at a protein concentration of 350 nM (see text). ND: not determined.

Observations quite similar to those with heavy metal ions have been reported with the metalloid, arsenic. Arsenic(III) compounds have been found to interfere *in vitro* with the oxidative refolding of three different disulfide-containing proteins; lysozyme and ribonuclease with four disulfide bridges and riboflavin-binding protein with nine disulfide bridges. Arsenicals at a micromolar concentration inhibited the oxidative refolding by formation of bi- and tri-dentate complexes with the cysteine residues of the chemically denatured reduced proteins [8]. A subsequent study showed that arsenite also interferes with the refolding of chemically denatured luciferase, which contains four free cysteine residues and no disulfide bridge [9]. Arsenite with an IC_50_ in the three-digit micromolar range is a considerably less effective inhibitor of *in vitro* protein refolding than heavy metal ions. However, the consequences of arsenite and heavy metals interfering with protein refolding *in vitro* are similar; in both cases, β-structured aggregates of inactive misfolded proteins are produced with an enhanced affinity for thioflavin-T [7,8]. The aggregation might not only be due to intermolecular hydrophobic interactions of metal-induced misfolded proteins, but might in part also be initiated by metal ions complexing with two polypeptide chains at the same time. The metal-induced aggregates (after gel filtration to remove uncomplexed metal ions) have a seeding capacity and promote the aggregation of refolding protein without the *de novo* addition of metal ions [9,48].

Xenobiotic heavy metals and metalloids other than those tested, as well as essential heavy metals in an overdose may also be expected to perturb protein folding. Indeed, it has been observed that the *in vitro* renaturation of chemically denaturated metal-free alcohol dehydrogenase is inhibited by Zn^2+^ in an overdose. The inhibition was suggested to be due to the blocking of an inactive intermediate in an incorrect conformation [49].

## 5. Heavy Metals and Metalloids Interfere with Protein Folding and Induce Protein Aggregation in Living Cells

As discussed above, certain heavy metals and metalloids potently inhibit protein folding *in vitro*. Recent work in yeast demonstrated that this mode of action also occurs in living cells [9,48,50]. *Saccharomyces cerevisiae* (budding yeast) cells accumulated aggregated proteins following exposure to equi-toxic concentrations of arsenite, cadmium and chromium (Cr(VI)) in the order As > Cd > Cr [9]. The *in vivo* potency of these agents to trigger protein aggregation probably depends on the efficiency of their cellular uptake/export and on their distinct modes of biological action. Arsenite-induced protein aggregation was shown to be concentration-dependent, and the aggregates contained a wide variety of proteins enriched in functions related to protein synthesis, metabolism and protein folding and stabilization. Notably, these aggregates are largely distinct from so-called processing bodies (P-bodies) and stress granules, structures known to form in response to arsenite exposure. Several chaperones co-sedimented with the aggregates, indicating that arsenite promotes protein misfolding *in vivo*. Indeed, arsenite was demonstrated to interfere with protein folding in living cells in two ways. Firstly, arsenite affected the folding of nascent proteins during the processes of translation, likely by acting on not yet folded segments of the polypeptide chain; secondly, it directly inhibited chaperone activity [9] (the chaperone(s) targeted remain unidentified for the time being). One probable target is the CCT (chaperonin-containing T) complex, since arsenite inhibits CCT activity *in vitro* [51] and several components of the CCT complex have been found among the arsenite-aggregated proteins [9]. Other putative targets include the ribosome-associated Hsp70s Ssb1p and Ssb2p, as well as cytosolic Ssa1p; these yeast chaperones contain surface-exposed reactive cysteine residues [52] and have been found among arsenite-aggregated proteins [9]. Thus, arsenite might directly bind to or modify critical cysteine residues and thereby inhibit the activity of these chaperones. In this respect, the ribosome-associated Hsp70s Ssb1p and Ssb2p are of particular interest, because a major fraction of the proteins that aggregate in yeast cells following arsenite exposure appear to be co-translational substrates of Ssb2p [50]. Hence, besides acting on nascent polypeptides during translation, arsenite may also target chaperones for inactivation and/or aggregation, thereby diminishing the overall folding capacity of the cell. Both mechanisms of arsenite action will lead to widespread protein misfolding and aggregation.

Arsenite-induced protein aggregation correlates with the toxicity of the metalloid [9]; however, it is not entirely understood how these aggregates affect cell viability. Toxicity could potentially be caused by the depletion of individual proteins that aggregate. However, the overlap between yeast proteins that aggregate during arsenite exposure and the arsenite sensitivity of the corresponding gene deletion mutants is poor, suggesting that inactivation/depletion of individual proteins by aggregation, i.e., by loss of function, is not a major toxicity mechanism. On the other hand, the proteins that aggregate upon arsenite exposure are enriched for multiple protein-protein interactions [50], suggesting that misfolded forms of these proteins might engage in extensive aberrant protein-protein interactions during exposure, thereby affecting cell viability. Consistent with this notion, *in vitro* data indicate that arsenite-induced protein aggregates, in a gain-of-function mechanism, act as seeds committing other, labile proteins to misfold and aggregate [9].

Widespread aggregation of nascent proteins is also observed in cadmium-exposed yeast cells [9,48,53]. In addition, cadmium has been shown to cause endoplasmic reticulum (ER) stress in yeast [54] and probably also in mammalian cells [55,56,57,58]. Neither arsenite nor mercury provoke any ER stress in yeast [54]. ER stress is characterized by an accumulation of unfolded proteins in the ER, which, in turn, activates the unfolded protein response (UPR). The UPR, which is operative in most eukaryotic cells from yeast to mammals, induces the expression of proteins that facilitate protein folding in the ER, thus mitigating the ER stress [59]. Yeast mutants that cannot induce the UPR are hypersensitive to cadmium, suggesting that toxicity might be a consequence of cadmium accumulation in the ER [54]. Whilst cadmium does not interfere with the formation of disulfide bonds in the ER, it appears to perturb calcium metabolism [54], a condition that may result in ER stress [60]. The mechanistic details of how cadmium causes protein misfolding in the cytosol and the ER remain to be elucidated.

Chromium (Cr(VI)) has been shown to trigger oxidative protein damage [61] and protein aggregation in yeast [9,10]. Whilst arsenite and cadmium directly interfere with protein folding, chromium induces protein aggregation by enhancing mRNA mistranslation. Mistranslation appears to be a primary cause of cellular chromium toxicity [10]. Although the mechanistic details of how chromium provokes mRNA mistranslation remain unknown, misincorporation of amino acids may result in misfolding and aggregation of the corresponding proteins. Neither arsenite nor cadmium cause mRNA mistranslation to any major extent [9,48].

To conclude, these recent studies in yeast demonstrated that heavy metals and metalloids interfere with protein folding in living cells through common, as well as distinct mechanisms, nascent or non-folded proteins being the prime targets. Quite likely, heavy metals and metalloids other than those already tested will perturb protein folding and manifest their toxicity through similar mechanisms.

## 6. How Cells Deal with Heavy Metal- and Metalloid-Induced Protein Misfolding and Aggregation

Protein quality-control systems monitor the correct functionality of the proteome and protect cells against the harmful accumulation of protein aggregates during environmental stress conditions, as well as during disease and ageing processes [11,20]. In response to heavy metal exposure, cells adjust their gene expression programs; (1) chaperone- and other heat-shock-response genes are activated, probably to enhance the folding capacity of the cell [62,63,64,65,66,67,68,69,70]; (2) proteasome-encoding genes are activated to enhance the protein degradation capacity of the cells [64,65,66,67,71,72,73]; and (3) the expression of genes encoding aggregation-prone proteins is downregulated, presumably to prevent excessive accumulation of harmful aggregates [50,74]. Failure to adjust the protein quality-control systems will result in increased metal/metalloid sensitivity [9,65,66,67,75].

Thermotolerance in yeast and bacterial cells has been shown to be based on chaperone-mediated protein rescue rather than the degradation of aggregated proteins [76,77]. Albeit chaperones and other heat-shock proteins are induced by heavy metals and metalloids, it is not clear to what extent the rescuing of proteins contributes to tolerance. Arsenite exposure results in enhanced expression of the disaggregating chaperone, Hsp104p, as well as other heat-shock proteins in yeast [9,65,70]; however, whilst yeast cells lacking Hsp104p are 100- to 1,000-fold more sensitive to heat than wild-type cells, Hsp104p provides only a two- to three-fold survival advantage during exposure to lethal concentrations of arsenite [70]. Likewise, Hsp104p is strongly induced by cadmium, but contributes little, if anything, to cadmium tolerance [70]. There are several ways to interpret these observations. From an aggregation point of view, aggregates produced by heat, on the one hand, and cadmium/arsenite, on the other hand, may be fundamentally dissimilar. From a toxicity point of view, the main lesion produced by arsenite and cadmium may not be accessible to Hsp104p (e.g., different compartments) or may not be a protein. From a chaperone point of view, arsenite and cadmium may directly inhibit Hsp104p or other chaperones required to restore folding homeostasis. The latter mechanism may account for the poor performance of Hsp104p in arsenite tolerance [70] and its inability to dissolve aggregates when this metalloid is present [9].

The capacity to degrade misfolded proteins is important during stress conditions and for protecting cells against a variety of protein misfolding disorders [78]. The ubiquitin-proteasomal system mediates the degradation of metal-induced protein aggregates and is clearly important for heavy metal tolerance in yeast: cadmium-treated cells enhance protein degradation rates via the ubiquitin-proteasome pathway [53]; arsenite-exposed cells increase proteasomal activity and clear the cytosol from arsenite-induced aggregates primarily via proteasomal degradation [9,79]; and yeast cells with diminished proteasomal activity are sensitized toward arsenite, cadmium and chromium [9,10,67,75,80]. Proteasomal activity can be controlled both at the transcriptional and post-translational level. For instance, yeast cells induce the expression of proteasome-encoding genes in response to arsenite exposure and, thus, enhance proteasomal activity [9]. Upon arsenite exposure, yeast appear to regulate proteasomal activity also at the post-transcriptional level [9]. The mechanistic details of this regulation are currently unknown. Mammalian cells induce the expression of the AIRAP protein in response to arsenite exposure. AIRAP interacts with the proteasome to enhance its stability and/or activity [71]. The mechanism of how AIRAP regulates the proteasome function is unclear.

Chelation and complexation is another strategy that cells utilize to deal with toxic metals and metalloids. Yeast exposed to arsenite, cadmium or chromium induce the expression of genes in the glutathione (GSH) biosynthesis pathway and accumulate high amounts of reduced GSH [65,66,81,82,83,84]. Amongst other mechanisms, GSH contributes to metal detoxification by complexing metal ions. Metal-GSH complexes are recognized by membrane-bound transporters that catalyze their export out of cells or their sequestration in an intracellular compartment [5,85]. In addition, GSH protects the proteome against arsenite-induced aggregation; yeast cells deficient in GSH biosynthesis accumulated more aggregated proteins than wild-type cells upon arsenite exposure, whereas cells with increased arsenite-chelating capacity accumulated fewer aggregates [86]. Thus, by complexing arsenite, GSH lowers the levels of free metalloid in the cytosol and prevents it from interacting with nascent proteins. Other metal-binding agents, such as metallothioneins (present in a wide range of organisms from bacteria to mammalian cells) or phytochelatins (present in plants and some fungi) are known to buffer metal ions via sequestration [1]. Thus, metallothioneins and phytochelatins act to keep the cellular concentration of free metal ions low and might therefore also prevent interactions between metals and nascent proteins.

It has been suggested that aggregation is an intrinsic property of all proteins and that proteins are only just soluble at the concentration levels at which they are expressed in the cell [87]. Accordingly, yeast proteins predicted to be aggregation-prone appear to be kept at concentrations below a critical threshold that would prevent their aggregation [74]. Moreover, the expression of the proteins that were found to aggregate in yeast cells following arsenite exposure was downregulated in the presence of the metalloid [50]. Whether downregulation of these proteins protects cells from excessive misfolding and aggregation remains to be demonstrated. Nevertheless, the data above suggests that cells may sense and signal disturbed protein homeostasis to the transcriptional machinery. The sensing and signaling mechanisms that control this response are currently unknown.

Another means to protect the intracellular environment from potentially toxic protein species is to sequester aggregated proteins to specific deposit sites. Yeast and mammalian cells relocate misfolded proteins that are ubiquitylated to a juxtanuclear quality-control compartment (JUNQ), where they are processed for degradation, whilst non-ubiquitylated proteins are diverted to peripheral insoluble protein deposits (IPOD) [88]. Whether heavy metal/metalloid-induced aggregates are sequestered to specific deposit sites, like JUNQ and/or IPOD, has not been explored as yet.

Finally, the most prominent heavy metal/metalloid tolerance mechanism is provided by a range of membrane-bound transporters that remove xenobiotic agents from the cytosol either by catalyzing their export out of the cell or by catalyzing their sequestration into intracellular compartments [1]. The activity of such export pathways decreases cytosolic levels of metals/metalloids and protects the intracellular environment from metal damage. Indeed, overexpression of an arsenite export protein in yeast has been shown to diminish intracellular arsenic levels and to prevent protein aggregation [9].

## 7. Metal-Induced Protein Aggregation and Protein Misfolding Disorders

Protein misfolding and aggregation are molecular hallmarks of several neurodegenerative and age-related disorders, including Alzheimer’s disease, amyotrophic lateral sclerosis and Parkinson’s disease. In these disease states, specific proteins adopt non-native conformations and aggregate. In addition, aberrant interactions between disease-associated and other cellular proteins might lead to extensive co-aggregation and loss of function of non-disease proteins [11,13]. There is accumulating evidence that heavy metals and metalloids may influence the aggregation properties of disease-associated proteins and promote certain neurodegenerative diseases through largely unknown mechanisms [14,15,16,17,18,19,89,90]. The mechanism of toxic action described here suggests that the deleterious effects of heavy metals and metalloids may result from short-term, direct interactions of metal ions with folding proteins. Additionally, long-term interactions of low concentrations of metal-induced seed aggregates may prompt other labile proteins to misfold and aggregate [9]. In mammals, these effects may become amplified with age when the efficiency of chaperone- and protease-dependent proteostasis mechanisms declines [91]. Remarkably, several yeast proteins that aggregate in response to arsenite have human homologues that are present in aggregates associated with protein folding disorders [50]. Hence, the effects of arsenic, as well as heavy metals described here might contribute to protein misfolding disorders.

## 8. Perspectives

It is becoming increasingly clear that heavy metals and metalloids profoundly affect protein homeostasis and cell viability by interfering with protein folding processes in living cells. This mechanism of toxicity does not only affect individual proteins, but also results in the formation and cellular accumulation of toxic protein aggregates. Chronic metal exposure is associated with age-related and neurodegenerative disorders caused by aberrant protein folding. A detailed understanding of the molecular mechanisms by which metals interfere with protein folding and promote protein aggregation *in vivo*, how aggregates impair cellular functions, how cells regulate the protein quality-control systems to protect against aggregate toxicity and how these processes are linked to disease pathologies may ultimately contribute to the development of new strategies for the prevention and treatment of protein misfolding disorders.
